# Convergent evolution of marine mammals is associated with distinct substitutions in common genes

**DOI:** 10.1038/srep16550

**Published:** 2015-11-09

**Authors:** Xuming Zhou, Inge Seim, Vadim N. Gladyshev

**Affiliations:** 1 Division of Genetics, Department of Medicine, Brigham and Women’s Hospital, Harvard Medical School, Boston, MA 02115, USA

## Abstract

Phenotypic convergence is thought to be driven by parallel substitutions coupled with natural selection at the sequence level. Multiple independent evolutionary transitions of mammals to an aquatic environment offer an opportunity to test this thesis. Here, whole genome alignment of coding sequences identified widespread parallel amino acid substitutions in marine mammals; however, the majority of these changes were not unique to these animals. Conversely, we report that candidate aquatic adaptation genes, identified by signatures of likelihood convergence and/or elevated ratio of nonsynonymous to synonymous nucleotide substitution rate, are characterized by very few parallel substitutions and exhibit distinct sequence changes in each group. Moreover, no significant positive correlation was found between likelihood convergence and positive selection in all three marine lineages. These results suggest that convergence in protein coding genes associated with aquatic lifestyle is mainly characterized by independent substitutions and relaxed negative selection.

It has been suggested that convergent evolution is characterized by convergent or parallel amino acid substitutions at specific amino acid sites in distant organisms^1^,^2^. For example, more than half of the substitutions (62% of 126 experimental substitutions) in two replicate populations of bacteriophage ϕX174 were parallel, and approximately 94% of candidate convergently evolved genes in a study of distant echolocating mammals (toothed whales and echolocating bats) contained parallel amino acid substitutions^3^,^4^. Given that these conclusions are typically drawn from experiments with animals and/or analyses of pairs of organisms that independently acquired a trait of interest^3^,^4^,^5^,^6^ and may be subject to strong artificial selection and random substitutions, it is of interest to examine the molecular basis for convergent adaptations in three or more independent natural groups.

One such opportunity is offered by the phenotypic convergence following the transition of mammals from terrestrial to aquatic environments, which occurred at least three times independently^7^. Extant marine mammals include more than 120 species belonging to three distinct mammalian orders: Carnivora (walruses, sea lions, and other pinnipeds), Cetacea (whales, dolphins, and porpoises), and Sirenia (manatees and dugongs). Cetaceans and sirenians originated approximately 50 million years ago in the early Eocene, while pinnipeds trace their ancestry back to late Oligocene^8^. Because of similar constraints imposed by an aquatic environment, marine mammals are characterized by obvious morphological convergence, such as a streamlined body shape with modified limbs (pinnipeds have front and hind limbs modified as flippers, whereas cetaceans and sirenians completely lost their hind limbs). In addition, most marine mammals present a respiratory system adapted for reduced oxygen consumption, which enables them to withstand hypoxia and prolong deep dives^8^. Despite a rich history of the field, specifically in the context of physiological adaptations of marine mammals, relatively little is known about the molecular changes that underlie convergent genetic adaptations associated with aquatic life. Several previous studies describing mutations in marine mammals have focused on cetaceans. For instance, Wang *et al.*^9^ reported that *HOXD12* is under Darwinian selection and associated with two episodes of cetacean forelimb reorganization. Mirceta *et al.*^10^ reported an adaptive molecular signature where elevated myoglobin (*MB*) net surface charge in cetaceans and pinnipeds is mechanistically linked with maximal myoglobin concentration. Very recently, Foote and colleagues^11^ performed genome sequencing of three species of marine mammals (the killer whale, walrus, and manatee) and interpreted the convergent evolution of marine mammals by counting parallel amino acid substitutions in their genomes. No apparent correlation between functional enrichment of genes with parallel substitutions and aquatic adaptation was found, with the observed parallel substitutions also detected throughout genomes of terrestrial taxa with no obvious phenotypic convergence^11^. Here, aided by whole genome alignments of a greater number of mammalian species and evolutionary model analyses, we reexamined the contribution of parallel substitutions to genomic convergence in the three distinct marine lineages and identified a subset of genes which may contribute to the aquatic phenotype.

## Results

### Most parallel substitutions are not unique to marine mammals

**Figure 1:**
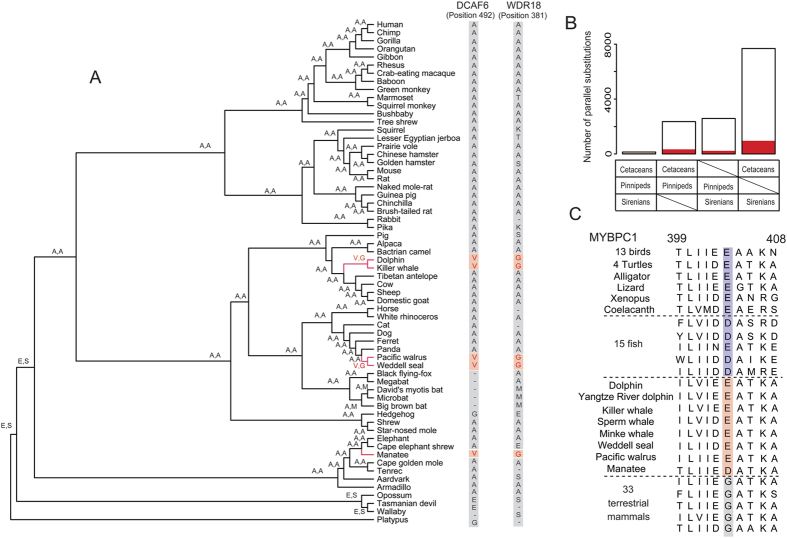
Parallel and unique substitutions in marine mammals. **(A)** Parallel substitutions in DCAF6 and WDR18 in marine mammals. We utilized genomic data of 5 marine mammals and 57 terrestrial mammals with completed genomes. Amino acid residues associated with each branch are based on reconstructed ancestor sequences at the corresponding positions in DCAF6 and WDR18. (**B**) Number of parallel substitutions along the branches of the three marine groups, or at least two marine mammal lineages, since they evolved from a terrestrial ancestor. Parallel substitutions unique to the indicated marine groups are shaded red. **(C)** Deduced partial amino acid sequence alignment of MYBPC1. The common substitution identified is located at amino acid position 404 of the human ortholog. The corresponding sites in terrestrial mammals are shown in gray, in marine mammals in orange, and in fish, birds, amphibians, and reptiles in blue.

To investigate molecular convergence, we first identified parallel amino acid residue changes in the three groups of marine mammals. A parallel substitution was defined as an amino acid change at the same position in marine mammals different from that of the ancestral node of each marine group, but identical in the three marine groups. Aided by human UCSC whole-genome multiple alignments, protein-coding sequences representing 5 marine and 57 terrestrial mammals were obtained, and ancestral sequences of each node in the phylogenetic tree were reconstructed (Fig. 1A, Supplementary Fig. S1). In total, 133 parallel amino acid residue substitutions in 132 genes were identified in all three marine groups, as well as 2,351, 7,684, and 2,579 parallel substitutions in cetaceans + pinnipeds, cetaceans + sirenians, and pinnipeds + sirenians, respectively (Fig. 1B, Supplementary Table S1).

Independent of the analysis of parallel substitutions, we considered all mammalian species in the data set and identified unique substitutions in marine mammals, which were defined as any amino acid residue at the same position in all three marine mammal groups that is neither found in the ancestral nodes with their respective terrestrial taxa nor in other terrestrial mammals. This scan identified 26 genes with unique amino acid changes in 8 marine mammals (Supplementary Table S2). Some of the unique changes in marine mammals are of interest because the genes are associated with phenotypic changes or physiological functions indicative of aquatic adaptation. For example, *MYBPC1* encodes a slow skeletal muscle isoform of myosin-binding protein C that supports muscle contraction by recruiting muscle-type creatine kinase to myosin filaments^12^,^13^. *KCNG4* encodes Kv6.3, which complexes with oxygen-sensitive Kv2 potassium channel subunits to regulate vascular tone^14^. *CPT2*-encoded protein controls fatty acid oxidation^15^, and mutations in *TMC8* are associated with an autosomal recessive genetic hereditary skin disorder in humans^16^.

If parallel substitutions represent a measure of genomic convergence, one may ask how many parallel substitutions are unique to groups of interest and whether the number of such substitutions exceeds that of control groups. Based on the parallel substitutions and unique amino acid changes identified in the three marine lineages, we found that the majority of substitutions were not unique to marine groups (i.e., they are also present in some terrestrial mammals) (Fig. 1B). In particular, of the 133 parallel substitutions in the three marine groups, only two (substitutions in *DCAF6* and *WDR18*) were unique to all marine mammals (Fig. 1A). For parallel substitutions in two marine groups, 10.5%, 11.4%, and 8.3% represented unique changes in cetaceans + pinnipeds, cetaceans + sirenians, and pinnipeds + sirenians, respectively (Fig. 1B). Thus, the majority of parallel substitutions in marine mammals also occur in terrestrial mammals. In addition, when we replaced one of the marine lineages with its terrestrial sister taxa, parallel substitutions in the three marine lineages still did not exceed the numbers in any two marine groups plus the control terrestrial taxa (Supplementary Fig. S2). Nevertheless, *DCAF6* and *WDR18* represent candidate genes for further functional investigation. DCAF6 functions as a ligand-dependent coactivator of nuclear receptors and interacts directly with androgen receptor (AR)^17^. The regulation of male skeletal integrity by AR signaling can be attributed to both osteoblasts and osteocytes^18^. *WDR18* encodes a WD-repeat protein that is highly conserved among vertebrate species and prior investigations have shown that WDR18 has an essential role in determination and regulation of zebrafish laterality^19^.

### Backward substitutions in marine mammals

Interestingly, the common amino acid residue change observed in marine mammalian MYBPC1 (Gly404Glu/Asp) is also found in fish, amphibians, reptiles, and birds (Fig. 1C, Supplementary Fig. S3). Since the function of the Ig domain of myosin-binding protein C, in which this residue is located (Supplementary Fig. S4), is not known, the specific role of this ‘backward’ substitution in marine mammals is unclear. Interestingly, a recent study found that mutations in *MYBPC1* are associated with distal arthrogryposis type 1, a disorder characterized by joint deformities that restrict movement in the hands and feet of humans^20^. In addition, the corresponding malgenic mutations result in a bent body curvature and decreased motor activity in zebrafish^21^. Thus, we hypothesize that parallel substitutions in MYBPC1 may be associated with limb development and skeletal muscle function in marine mammals.

The site altered in CPT2 of marine mammals (Arg/His37Gln/Lys) is also changed in fish (Arg37Lys/Ser) and the semi-aquatic platypus (Arg37Lys) (Supplementary Fig. S3). Carnitine palmitoyltransferase II (*CPT2*) plays important roles in fatty acid oxidation and energy metabolism. Mutations in *CPT2* are associated with carnitine palmitoyltransferase II deficiency^22^, disabling the use of certain lipids for energy generation, particularly during fasting. A recent study found that *CPT2* gene expression is significantly increased in the liver of rabbits fed Omega-3 fatty acids^23^. Given the similar nutrient sources of marine mammals, platypus, and some fish, we surmise that the parallel substitution in CPT2 may correlate with dietary preferences in the aquatic environment.

### Identification of genes associated with aquatic adaptation by evolutionary model analyses

Since the majority of parallel substitutions are not unique to marine mammals and it is generally thought that orthologs in mammals are functionally conserved, it is doubtful that the large number of non-unique parallel substitutions and/or rare unique amino acid changes significantly contributes to phenotypic and physiological convergence in marine mammals. Thus, alternative methods are necessary. To identify genes that may be associated with an aquatic lifestyle, we next considered evolutionary models and examined whether candidate genes are enriched with parallel substitutions.

**Figure 2:**
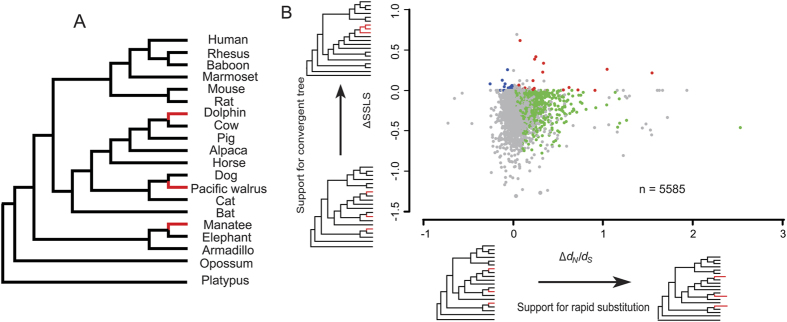
Evolutionary model analyses of genomic convergence of marine mammals. **(A)** Phylogenetic relationship of mammals used to identify rapidly and convergently evolved genes. Branches representing independent evolution of marine mammal lineages, for which tests for rapid substitution, likelihood convergence, and positive selection were performed, are colored red. Branches of the terrestrial taxa control set, are cow, dog, and elephant, respectively. **(B)** Distribution of Δ*d*_*N*_*/d*_*S*_ and ΔSSLS in 5,585 orthologs (n). Loci identified by ΔSSLS, Δ*d*_*N*_/*d*_*S*_ and both methods are shown in blue, green and red, respectively, and ΔSSLS shown here is estimated from an alternative topology (H_A_, tree 1) as opposed to a common species tree (H_O_) (indicated by red branches to the left and below the plot).

Taking into account completeness of available genomes and the number of species feasible for the scale of such analyses, the phylogenomic data consisted of 5,585 protein-coding orthologs from 20 mammals, representing all four major mammalian clades (Fig. 2A). We selected three marine mammals (the common bottlenose dolphin, walrus, and manatee; which represent cetaceans, pinnipeds, and sirenians, respectively) to ensure that the elevated substitution rate and sitewise log-likelihood support measured in the evolutionary analyses were due to inter-order substitutions rather than substitutions within each marine group.

We first examined genes with accelerated rates of amino acid substitutions in aquatic mammals (measured by the ratio of nonsynonymous to synonymous substitutions, *d*_*N*_*/d*_*S*_) using the branch model^24^. For each gene, this model assigns a *d*_*N*_*/d*_*S*_ value to a ‘foreground branch’ (marine mammals) and another *d*_*N*_*/d*_*S*_ value to a ‘background branch’ (terrestrial mammals). The branch model (non-neutral) is compared to a null model (neutral model with single estimated *d*_*N*_*/d*_*S*_), an LRT (likelihood ratio test) applied to gauge the likelihood of the evolutionary models. Genes with significantly positive Δ*d*_*N*_*/d*_*S*_ (*d*_*N*_*/d*_*S*_ being larger in marine mammals than in terrestrial mammals) were defined as rapidly evolving genes in marine mammals. This analysis yielded 907 genes (16.2%, LRT *P *≤ 0.05; 142 genes after multiple testing adjustment) with significantly elevated protein sequence substitution rate in marine lineages compared to terrestrial mammals (Fig. 2B, Supplementary Figs S5 and S6).

To identify sequence convergence between marine mammal lineages, we employed a maximum likelihood (ML) approach^4^,^25^, hereafter termed likelihood convergence analysis. In this method, we examined each amino acid along a given gene alignment (CDS) and measured its fit (sitewise log-likelihood support; SSLS) to the commonly accepted species tree (termed H_0_) and to nine alternative topologies in which marine taxa were ‘forced’ into erroneous monophyletic clades (representing the convergence hypothesis; see Methods). The likelihood convergence analysis revealed 441 genes (7.9%, indexed by ΔSSLS and denoted likelihood convergence genes; 116 genes if ΔSSLS from all nine alternative hypotheses supported the convergence) with significant likelihood support for the grouping of marine mammals as opposed to the distribution based on the canonical species tree (Supplementary Figs S7–S9).

### Aquatic adaptation genes are characterized by neither parallel substitutions nor congruent positive selection

**Figure 3:**
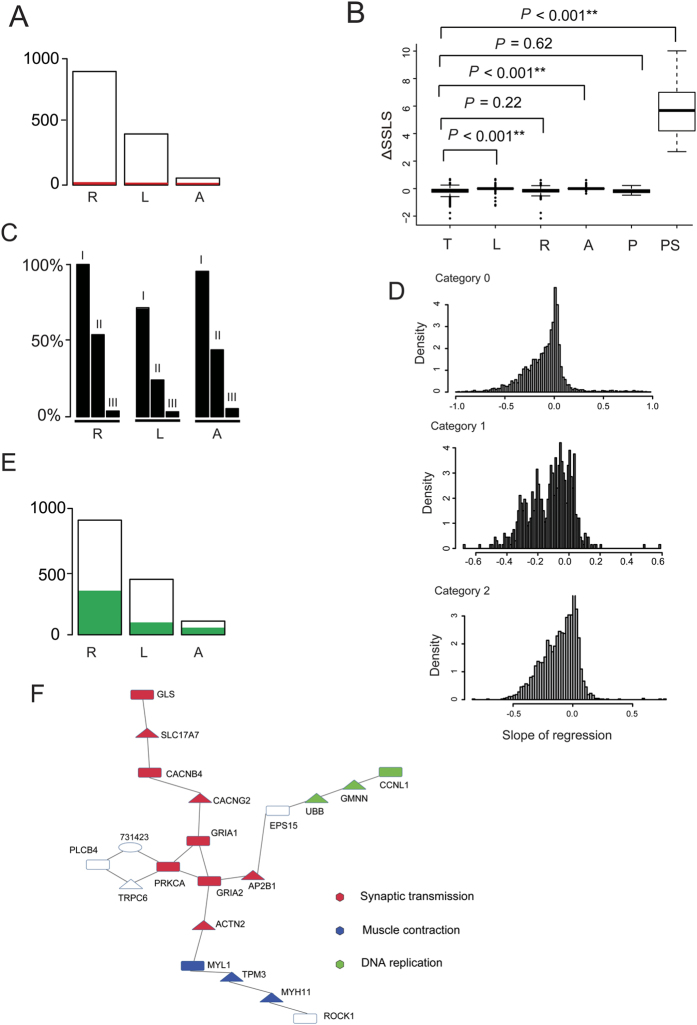
Statistics of rapidly evolving genes and likelihood convergence genes, and their functional enrichment. **(A)** Percentage of genes with parallel substitutions in rapidly evolving genes (R), likelihood convergence genes (L) and 70 aquatic adaptation genes (A). Red region denotes the proportion of parallel substitutions in marine mammals. (**B**) Statistics of average ΔSSLS of all orthologs (T), rapidly evolving genes (R), likelihood convergence genes (L), 70 aquatic adaptation genes (A), genes containing parallel substitutions (P), and parallel substitution sites (PS). (**C**) Percentage of genes with unique amino acid changes in rapidly evolving genes (R), likelihood convergence genes (L) and 70 aquatic adaptation genes (A). Unique amino acid changes were classified into those changing in all three lineages of marine mammals (III), in any two of the three marine lineages (II) and in single lineages of marine mammals (I). (**D**) The coefficient (slope) for locus-wise regressions between sitewise support for convergence and sitewise *d*_*N*_*/d*_*S*_ for sites under purifying selection (Category 0), neutral site (Category 1), and sites under diversifying selection (Category 2) are plotted. In each plot, there are loci showing negative relationship, characterized by slopes significantly below zero, and loci showing positive relationship, with slopes greater than zero. (**E**) Percentage of positively selected genes in rapidly evolving genes (R), likelihood convergence genes (L) and 70 aquatic adaptation genes (A). Positively selected genes were identified in cetaceans, pinnipeds, and sirenians, respectively. (**F**) Enriched network of 70 aquatic adaptation genes. Candidate genes are indicated by grey filled squares, missing genes by white filled triangles, and metabolites by white filled circles. Gene ontology categories are represented by Synaptic transmission in red, DNA replication in green, and Muscle contraction in blue.

Having identified putative convergent genes in marine mammals by evolutionary model analysis, we assessed the prevalence of parallel substitutions in the 20-mammal dataset. Using the commonly accepted ‘species tree’ shown in Fig. 2A^26^,^27^,^28^,^29^ and a likelihood method based on ancestral sequence reconstruction^30^, we identified 44 parallel substitutions in 38 genes in all marine mammals. As revealed by the aforementioned results for 62 mammals, the majority of parallel substitutions were not unique to each combination of two or three marine mammals (Supplementary Fig. S10). Similarity, only 14 rapidly evolved genes (1.5%) contained parallel substitutions (2.8% after multiple testing adjustment) and 6 likelihood convergence genes (1.4%) contained parallel substitutions in marine mammals (Fig. 3A). Of the 116 likelihood convergence genes (supported by ΔSSLS from 9 alternative hypotheses), only 3 genes contained parallel substitutions in marine mammals. We further examined the relative abundance of parallel substitutions in 70 genes (hereafter denoted as candidate aquatic adaptation genes), which received evidence from both elevated substitutions rate and likelihood scores support (with Δ*d*_*N*_*/d*_*S*_ > 0, LRT *P *≤ 0.05 and ΔSSLS > 0, *P *≤ 0.05) (Supplementary Table S3); only 5.7% of the 70 genes had parallel substitutions (Fig. 3A). It is worth noting that ΔSSLS values of parallel substitutions were significantly greater than the average ΔSSLS of all orthologs (*P *≤ 0.01, Student’s t-test), suggesting that most of the parallel substitutions supported the convergence hypothesis rather than species tree. However, there was no significant difference in average ΔSSLS between genes containing parallel substitutions and total orthologs (*P *≥ 0.05, Student’s t-test) (Fig. 3B).

We next searched for unique amino acid residue changes in all three marine groups, any two marine groups and single marine groups. Less than half of the rapidly evolving genes and likelihood convergence genes had unique changes in all three or any two marine groups (with less than 10% in all three marine groups). Strikingly, most of the genes (99.7% of rapidly evolving genes and 69.2% of likelihood convergence genes) contained distinct changes in each group of marine mammals (Fig. 3C). A similar trend was observed for the 70 candidate aquatic adaptation genes (Fig. 3C, Supplementary Table S4). These data show that candidate genes for convergent evolution of aquatic mammals are primarily characterized by distinct amino acid changes in each marine group.

Previous studies suggested that sequence convergence is a consequence of natural selection^4^. Of the 907 rapidly evolving genes in marine mammals, only 13 genes (1.4%) had a nonsynonymous to synonymous substitution (*d*_*N*_*/d*_*S*_) ratio greater than one, indicating that the elevated nonsynonymous to synonymous substitutions rate in marine mammals reflects reduced purifying selection rather than positive selection (Supplementary Fig. S6). We further investigated whether the elevated *d*_*N*_*/d*_*S*_ in marine mammals was caused by the sitewise log-likelihood support (ΔSSLS). For each ortholog, we fitted the linear relationship between ΔSSLS and the corresponding sitewise *d*_*N*_*/d*_*S*_ in marine lineages and classified sites into three categories, i.e. evolved under purifying-, neutral-, or diversifying- (positive) selection in marine mammals (Fig. 3D). This effort revealed that the majority of genes presented a negative correlation between sitewise *d*_*N*_*/d*_*S*_ and support for convergence (67.2%, 80.0%, and 75.4% of genes under purifying selection, neutrally, and diversifying selection, respectively). In particular, of 982 genes under diversifying selection with significant correlation between sitewise ΔSSLS and sitewise *d*_*N*_*/d*_*S*_, only 22 genes (2.2%) showed positive correlation, indicating that sitewise convergence in marine mammals is not achieved by positive selection.

We further considered individual marine lineages. Positively selected genes (PSGs) in single marine lineage, i.e., cetaceans, pinnipeds, and sirenians, were determined using the branch site model^31^, respectively. Rapidly evolving genes contained a total of 350 PSGs (38.6%), likelihood convergence genes contained 49 PSGs (11.1%) and, in addition, half (50%, 35 genes) of the 70 candidate aquatic adaptation genes were PSGs (Fig. 3E). These data suggest that candidate aquatic adaptation genes (rapidly evolving genes or likelihood convergence genes) are co-influenced by lineage-specific selection with amino acid changes at distinct sites, a phenomenon previously observed for sodium channels associated with convergent evolution of electric organs in fish^32^.

### Functions of candidate aquatic adaptation genes

The specific roles of the candidate genes associated with aquatic adaptation require future functional analyses. However, insights can be gained by functional gene ontology (GO) enrichment analysis. Analysis of the 70 candidate genes with elevated protein sequence substitution rates and likelihood support revealed enrichment of GO terms such as synaptic transmission (GO:0007268, *P *= 1.90 × 10^−4^, Fisher’s exact test) and transmission of nerve impulse (GO:0019226, *P *= 5.10 × 10^−4^, Fisher’s exact test) (*PRKCA*, *GRIA2*, *GRIA1*, *LIN7C*, *CACNB4*, *NOVA1*, *PARK7*) (Supplementary Table S5). The corresponding KEGG pathways over-represented among the candidate aquatic adaptation genes were long-term depression (*P *= 3.70 × 10^−4^, Fisher’s exact test) and long-term potentiation (*P *= 3.50 × 10^−4^, Fisher’s exact test) (Supplementary Table S6). In agreement, pathway analyses revealed a cellular network comprising 20 genes, including 10 of our candidate genes, centered on synaptic transmission and muscle contraction (Fig. 3F). Nerve activity is a major control mechanism of the muscle fiber type profile, and multiple signaling pathways have been implicated in activity-dependent changes of muscle fibers^33^. For example, *MYL1* encodes fast skeletal muscle myosin alkali light chains^34^. Four genes (*PRKCA*, *PLCB4*, *ROCK1*, *MAPK10*) in the Wnt signaling pathway were also present in the candidate aquatic adaptation gene list (*P *= 0.007, Fisher’s exact test) (Supplementary Table S6). Altered expression of genes associated with the Wnt pathway has been implicated in metabolic and structural transformation of Weddel seal skeletal muscle from a strictly terrestrial lifestyle as a pup to an aquatic lifestyle adapted for deep dives as adult animals^35^.

Candidate aquatic adaption genes were also significantly enriched for the terms mRNA stabilization (GO:0048255, *P *= 2.80 × 10^−4^, Fisher’s exact test) and RNA stabilization (GO:0043489, *P *= 2.80 × 10^−4^, Fisher’s exact test) (*HNRNPD*, *PABPC1*, *YBX1*). In response to hypoxic challenge a series of cellular responses are initiated in mammalian cells, triggering adaptive processes including changes in cell division, survival, motility, or differentiation^36^. Hypoxia markedly decreases total *de novo* transcription and, accordingly, several RNA-binding proteins (RBPs) have been found to affect the stability of many specific mRNAs. For example, accelerated mRNA decay is observed in response to hypoxia, and this process involves decay-promoting RBPs such as HNRNPD and PABPC1^37^. The mRNA–protein complexes transition into stress granules, highly specialized cytoplasmic structures and sites of mRNA storage that facilitate translational reprogramming^38^. A recent study demonstrated a role for *YBX1* in stress granule formation and tumor progression^39^. Taken together, the candidate aquatic adaptation genes encompass a plausible range of functional categories associated with marine lifestyles.

## Discussion

Our whole genome analyses revealed that parallel substitutions are widespread in marine mammals, consistent with a recent study by Foote and colleagues^11^. However, the majority of parallel substitutions are not unique to marine mammals. These findings do not rule out that parallel substitutions contribute to functional and phenotypic convergence of marine mammals. As the number of taxa increases, the likelihood of finding another homoplasious substitution in a terrestrial taxon increases as well. It should be appreciated that it is currently difficult to examine whether such signatures represent an unrelated homoplasy (if molecular convergence really has occurred amongst focal taxa), or a ‘true negative’ (all homoplasies have occurred neutrally and there is no molecular convergence). Here, by considering different classes of substitutions and sitewise ∆SSLS, we propose that rapidly evolving genes and sequence convergence in marine mammals are predominantly characterized by independent rather than parallel substitutions.

Two lines of evidence from experimental studies support this hypothesis. For example, the transfer of three related bacteriophage species to a novel environment revealed a high rate of parallel genetic evolution at orthologous nucleotide and amino acid residues within, but not between, species^40^. Similarly, a study involving *Pseudomonas aeruginosa* demonstrated that antibiotic resistance and pathogen fitness in individual isolates stemmed from parallel substitutions in four antibiotic resistance genes coupled with distinct substitutions in more than hundred other genes^41^,^42^. Following submission of our manuscript, two studies were published arguing that there is no excess of (parallel) convergence between echolocating mammals^43^,^44^. They demonstrated that methods solely based on sitewise likelihood convergence are not adequate for measuring genomic convergence^44^. This is consistent with our data and hypothesis, as non-unique parallel substitutions could be generated by chance in any pair of mammals following phylogenetic distance. Therefore, a combination of different evolutionary models, for example, the use of substitution rate and likelihood scores, as employed in the present study, may be necessary for future studies of convergent evolution.

It should be noted that multiple factors, including genome size and complexity, may influence whether parallel mutations occur at the same nucleotide positions or in the same gene (or complex locus)^45^. In the case of marine mammals, there may be two additional factors that result in distinct substitutions in common genes. First, terrestrial mammals returned to the sea at different evolutionary periods and, thus, under different genomic and environmental contexts. Second, it is clear that the adaptation to aquatic environments required multiple episodes of phenotypic convergence and likely involved multiple potentially adaptive mutations. Third, convergence generally involves four types of molecular evolution^46^. Although sequence convergence is the easiest to detect, other mechanisms (functional, mechanistic and structural convergence) may also play critical roles in the convergent evolution of marine mammals. Two types of mutation changes which may correlate with the aquatic adaptation of marine mammals have not been interrogated in the present study: mutations in distinct genes associated with the same pathway and mutations in *cis* regulatory regions. For example, distantly-related electric fish show both sequence convergence^32^ and mechanistic convergence in the form of common gene expression patterns enabling the development of electric organs^47^. In addition, regulatory mutations in *PITX1* show signatures of positive selection in pelvic-reduced populations^48^. Future studies, for example functional or genomic analyses of non-coding regions, of marine mammals may complement our present study and provide further insights into the genomic basis of aquatic adaptations in mammals.

## Methods

### Data collection

Coding sequences (CDS) of 62 mammals were obtained from the human UCSC 100 species whole-genome multiple alignment (build 04.15.2014)^49^. For genes with many reference sequences, the longest coding sequence was chosen. To confirm common changes in cetaceans that had not been included in the human UCSC 100 species, coding sequences of the Yangtze River dolphin (*Lipotes vexillifer*, assembly accession: GCA000442215.1), common minke whale (*Balaenoptera acutorostrata*, assembly accession: GCA000493695.1), and sperm whale (*Physeter catodon*, assembly accession: GCA000472045.1) were retrieved from NCBI by parsing genomic annotation files. In order to conduct evolutionary model analyses, coding sequences from species shown in Fig. 2A were interrogated. Cow (*Bos taurus,* UMD3.1), dog (*Canis lupus familiaris*, CanFam3.1), nine-banded armadillo (*Dasypus novemcinctus*, Dasnov3.0), human (*Homo sapiens*, GRCh38), rhesus (*Macaca mulatta*, MMUL 1.0), baboon (*Papio anubis*, PapAnu2.0), marmoset (*Callithrix jacchus*, C_jacchus3.2.1), African elephant (*Loxodonta africana*, Loxafr3.0), mouse (*Mus musculus*, GRCm38.p3), (bottlenose) dolphin (*Tursiops truncatus*, turTru1), pig (*Sus scrofa*, Sscrofa10.2), alpaca (*Vicugna pacos*, vicPac1), horse (*Equus caballus*, EquCab2), cat (*Felis catus*, Felis_catus_6.2), large flying fox (*Pteropus vampyrus*, pteVam1), platypus (*Ornithorhynchus anatinus*, OANA5) and gray short-tailed opossum (*Monodelphis domestica*, monDom5) were downloaded from Ensembl (Release 77). (Florida) manatee (*Trichechus manatus latirostris*) (Assembly accession: GCA000243295.1) and (Pacific) walrus (*Odobenus rosmarus divergens*) (Assembly accession: GCA000321225.1) sequences were obtained from NCBI Genomes.

### Identification of parallel amino acid substitutions

To identify parallel amino acid changes in marine mammals, the phylogenetic tree of 62 mammals used for reconstruction of ancestral sequences was trimmed from the original tree, with branch length from the human UCSC 100 species whole-genome multiple alignment. The ancestral sequences for each node were reconstructed with the help of FastML 3.1, which employs maximum likelihood algorithms and an empirical Bayesian approach taking into account the rate variation among sites^30^. We allowed FastML 3.1 to estimate the branch length of the phylogenetic tree for each gene when the ancestral sequences were reconstructed using the set of 20 mammals. The orthologous relationship of 51 genes in three other mammals was determined by best hit in local BLAST^50^, and protein-coding gene sequences were aligned with MUSCLE^51^. Amino acid changes of interest were manually validated.

### Evolutionary model analysis pipeline

In the evolutionary analyses, coding sequences (CDS) of individual genes from the human genome were used to query the other 19 genomes (listed in the Data collection section above and shown in Fig. 2A) for homologous sequences using reciprocal BLAST. Prior to using human genes to query genomes, we discarded sequences in which the CDS contained less than 150 nucleotides and CDS length was not divisible by 3. For genes with alternatively spliced transcripts in the human genome, the CDS with the greatest number of nucleotides were used in further analyses. Only hits with a BLAST e-score less than 1 × 10^−5^ were considered orthologs, and for cases in which there were multiple BLAST hits, the longest hit was chosen. Orthologous protein-coding genes from 20 mammals were aligned according to their translated amino acids using TranslatorX^52^ and MUSCLE^51^. PAL2NAL^53^ was used to identify conserved blocks for subsequent analyses. All analyses were run on the Orchestra supercomputing cluster supported by the Harvard Medical School Research Information Technology Group.

### Identification of rapidly evolving genes

To identify heterogeneous rates of protein evolution in marine mammals and terrestrial mammals, the branch model^24^ in the program *codeml,* available in the PAML package^54^, was used to fit each alignment and estimate differences in *d*_*N*_*/d*_*S*_ within the tree. The branch site model^31^ was used to detect positively selected genes in each marine lineage, respectively. A false discovery rate (FDR, Bonferroni method) correction was applied to account for multiple hypothesis testing. At least three rounds of analyses were performed to make sure that rapidly evolving genes were not affected by different start *d*_*N*_*/d*_*S*_ values (from 0.8 to 1.5).

### Detection of likelihood convergence genes

To detect signatures of molecular convergence, we fitted the orthologous CDS alignment data to a null model (H_0_, species tree) and an alternative model (H_A_, the constraint of monophyly of marine mammals). Next, we used the mean ΔSSLS (sitewise log-likelihood support) of all sites in a gene to index the strength of support for convergence^4^. This method directly compares the goodness-of-fit of each gene to a pair of phylogenetic trees under a given model. SSLS for each gene alignment was estimated by RAxML v7.4.8^55^, and ΔSSLS in the present study was calculated as ΔSSLS = lnL (H_A_) - lnL (H_0_). For example, genes with a better model fit to H_A_ (supporting convergence) will have positive ΔSSLS scores, whereas genes with a better fit to species tree (H_0_) will have negative ΔSSLS scores. We further estimated significance of convergence by performing analyses on simulated datasets. Fifty genes were randomly chosen to represent the alignment length and heterogeneity of our data. The simulations were carried out using Seq-Gen 1.3.3^56^, with 50 replicates. The ΔSSLS values of simulated data were collected and used to estimate their stepwise empirical cumulative density function (cdf), with linear interpolation^4^. The cumulative probability of the observed ΔSSLS under the null distribution was calculated, and we defined *P*, the significance of an observed site ΔSSLS comparison of the species topology H_0_, and an alternative topology H_A_ as *P *= 1-cdf(ΔSSLS_HA-H0_). The substitution model for each locus was determined by ProtTest 3.4^57^.

To gauge robustness of the results, we implemented WAG + GAMMA models of amino acid substitution instead of the JTT + GAMMA model in the model fit of sequence convergence. The substitution model did not have significant impact on the convergent genes in the present study. In the comparison of alternative models (convergent tree) and null model (species tree), we introduced 9 trees representing different topologies, but all supported the monophyly of marine mammals (Supplementary Fig. S7). The data presented in this study include genes identified from any pair of hypothesis tests between alternative tree and species tree (with defined *P* less than 0.05). We also used the average ΔSSLS values from 9 hypothesis tests to detect the correlation between likelihood convergence and adaptive selection.

### Sitewise selection pressure and clade model C

To estimate the sitewise *d*_*N*_/*d*_*S*_ (ω), the codon models M7 (the null model, with F3 × 4 frequencies) and M8 (sitewise selection)^58^ were fitted to each gene using *codeml* in PAML package^54^. We then considered the sitewise *d*_*N*_/*d*_*S*_ estimate only from loci where the M8 model was favored. Subsequently, we fitted the clade-specific Clade Model C (and null model M1a)^59^,^60^ and estimated *d*_*N*_/*d*_*S*_ on the hypothesized clade of marine mammals. In this model, three separate *d*_*N*_*/d*_*S*_ ratios were estimated in marine mammals; ‘category 0, denoted ω_0_, for sites under purifying selection where 0< ω <1; ‘category 1’, denoted ω_1_, for sites evolving neutrally, where ω = 1; and ‘category 2’, denoted ω_2_, for sites under diversifying selection, in which ω is free and can be larger than 1. In this model, the three ω ratios (ω_0_, ω_1_ and ω_2_) were calculated by ML and the Bayes empirical Bayes (BEB) posterior probabilities and we treated sites under purifying selection, neutrally, diversifying (positive) selection with a BEB posterior for each category >0.5.

Gene enrichment analyses were performed using DAVID (Database for Annotation, Visualization and Integrated Discovery)^61^ and R Spider^62^. All gene groups are potentially informative despite lower rankings and serve to guide biological interpretation^63^. Enriched Biological Process (BP) and Molecular function GO terms can be found in Supplementary Table S5 and KEGG pathway analysis can be found in Supplementary Table S6.

## Additional Information

**How to cite this article**: Zhou, X. *et al.* Convergent evolution of marine mammals is associated with distinct substitutions in common genes. *Sci. Rep.* **5**, 16550; doi: 10.1038/srep16550 (2015).

## Supplementary Material

Supplementary Information
