# Supplementary material for: Convergent evolution of marine mammals is associated with distinct substitutions in common genes
Source: Sci Rep. 2015 Nov 9;5:16550. doi: 10.1038/srep16550 (PMC4637874; doi:10.1038/srep16550)
Supplement: Supplementary Information [file srep16550-s1.pdf]

Supplementary Information

Convergent evolution of marine mammals is associated with distinct substitutions in common genes

Xuming Zhou, Inge Seim, Vadim N. Gladyshev

This file contains Supplementary Figures S1-S10 and Supplementary Tables S1-S6

Supplementary Figures

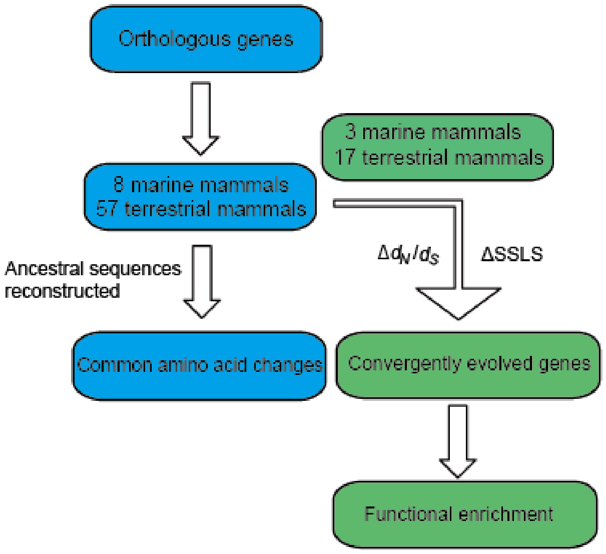

Supplementary Figure S1. Analysis pipeline in the present study. To identify common sequence changes in marine mammals, we utilized genomic data for 8 marine and 57 terrestrial mammals with completed genomes (shown in blue). To identify rapidly and convergently evolved genes, we subjected 3 marine and 17 terrestrial mammals to $\Delta d_N/d_S$ and ΔSSLS tests (shown in green).

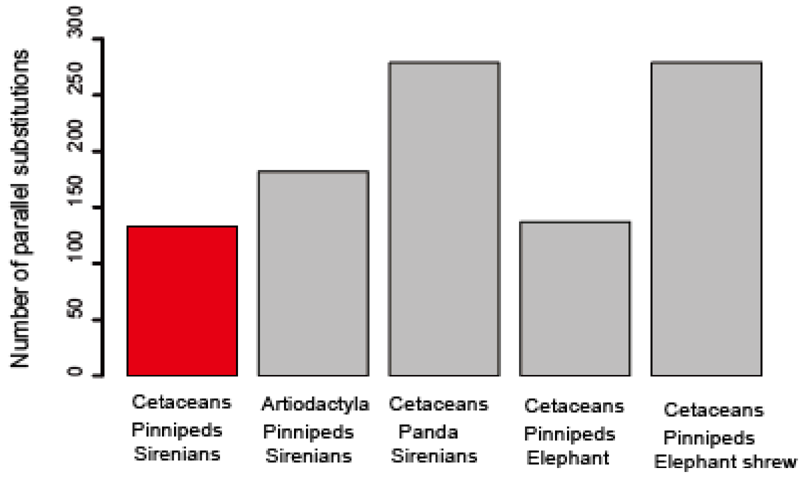

Supplementary Figure S2. Number of parallel substitution in the three marine lineages and in the combination of any two marine lineages with their sister terrestrial taxa.

MYBPC1

Species	399–408
Tetraodon	F L V I D D A S R D
Takifugu	F L V I D D A S R E
T._flavidus	F L V I D D A S R E
Oreochromis	F L V I D D A S R D
Neolamprologus	F L V I D D A S R D
Haplochromis	F L V I D D A S R D
Maylandia	F L V I D D A S R D
Pundamilia	F L V I D D A S R D
Oryzias	Y L V I D D A S R E
Xiphophorus	Y L V I D D A S K D
Gasterosteus	F L V I D D A S R E
Gadus	I L I I N E A T K E
Danio	W L I I D D A S K D
Astyanax	W L I I D D A I K E
Lepisosteus	I L I I D D A M R E
LepWed	I L I I D E A T K A
Tursiops	I L V I E E A T K A
Lipotes	I L V I E E A T K A
BalAcu	I L V I D E A T K A
Odobenus	I L I I E E A T K A
Trichechus	T L I I E D A T K A
Orcinus	I L V I E E A T K A
Physeter	I L V I D E A T K A
Ailuropoda	I L I I E G A T K A
Bos	I L I I D G A T K A
Callithrix	I L I I E G A T K A
Camelus	I L V I E G A T K A
Canis	I L I I E G A T K A
Capra	I L V I E G A T K A
Cavia	I L V I E G A T K A
Ceratotherium	I L I I E G A T K A
Chinchilla	I L I I E G A T K A
Chlorocebus	F L I I E G A T K A
Chrysochloris	T L I I E G A T K A
Condylura	F L I I E G A T K S
Cricetulus	I L I I E G A T K A
Dasypus	T L I I E G A T K A
Echinops	I L I I E G A T K A
Elephantulus	T L I I E G A T K A
Eptesicus	T L I I E G A T K A
Equus	I L I I E G A T K A
Erinaceus	I L V I E G A T K A
Felis	I L I I E G A T K A
Gorilla	I L I I E G A T K A
Heterocephalus	T L I I D G A A K A
Homo	I L I I E G A T K A
Jaculus	I L I I E G A T K A
Loxodonta	I L I I E G A T K A
Macropus	I L I I D G A T K A
Macaca	F L I I E G A T K A
Mesocricetus	I L I I E G A T K A
Microtus	T L I I E G A T K A
Mus	T L I I E G A T K A
Monodelphis	T L I I E G A T K A
Mustela	I L V I E G A T K A
Myotis	T L I I E G A T K A

CPT2

Species	34–43
Haplochromis	Y L H Q S V V P S M
Lepisosteus	Y L H K S I V P T M
Maylandia	Y L H Q S V V P S M
Neolamprologus	Y L H Q S V V P S M
Pundamilia	Y L H Q S V V P S M
Takifugu	Y L H Q S V V P T T
Physeter	Y L Q L S I V P T M
LepWed	Y L Q L S I V P T M
Lipotes	Y L Q L S I V P T M
Odobenus	Y L Q L S I V P T M
Tursiops	Y L Q L S I V P T M
BalAcu	Y L Q L S I V P T M
Trichechus	Y L Q S S I V P T M
Orcinus	Y L Q L S I V P T M
Ailuropoda	Y L Q R S I V P T M
Bos	Y L Q R S I V P T M
Callithrix	Y L Q R S I V P T M
Camelus	Y L Q R S I V P T M
Canis	Y L Q R S I V P T M
Cavia	F L Q H S I V P T M
Ceratotherium	Y L Q R S I V P T M
Chinchilla	Y L Q H S I V P T M
Chlorocebus	Y L Q R S I V P T M
Chrysochloris	Y L Q R S I V P T M
Condylura	Y L H R S I V P T M
Cricetulus	Y L Q H S I V P T M
Dasypus	Y L Q R S I V P T M
Elephantulus	Y L Q R S I V P T M
Eptesicus	Y L Q R S I V P T M
Equus	Y L Q R S L V P T M
Erinaceus	Y L Q R S I V P T M
Felis	Y L Q R S I V P T M
Heterocephalus	Y L H H S I V P T M
Homo	Y L Q R S I V P T M
Jaculus	Y L Q R S I V P T M
Macropus	Y L H H S I V D T M
Macaca	Y L Q R S I V P T M
Mesocricetus	Y L Q H S I V P T M
Microtus	Y L Q H S I V P T M
Mus	Y L Q H S I V P T M
Monodelphis	Y V H R S V I P T L
Mustela	Y L Q R S I V P T M
Myotis	Y L Q R S I V P T M
Nomascus	Y L Q R S I V P T M
Ochotona	Y L Q R S I V P T M
Octodon	Y L Q H S I V P T M
Ornithorhynchus	Y L H K S T V N T L
Orycteropus	Y L Q R S I V P T M
Otolemur	Y L Q H S I V P T M
Ovis	Y L Q R S I V P T M
Pan	Y L Q R S I V P T M
Papio	Y L Q R S I V P T M
Pongo	Y L Q R S I V P T M
Pteropus	Y L H R S V V P T M
P. vampyrus	Y L H R S V V P T M
M. mulatta	Y L Q R S I V P T M

Supplementary Figure S3. Non-synonymous amino acid substitutions found in marine mammals and their positions in *CPT2* and *MYBPC1*. Unique changes in marine mammals are shown in red, and changes in fish are in blue.

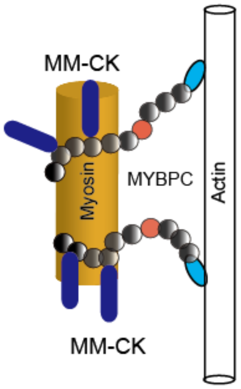

Supplementary Figure S4. Schematic representation of MYBPC protein geometry and its functional association with MM-CK (muscle-type creatine kinase). Domains are represented as black circles (with the domain containing the common change in marine mammals in red). The myosin filament backbone is shown in yellow, and MM-CK in blue.

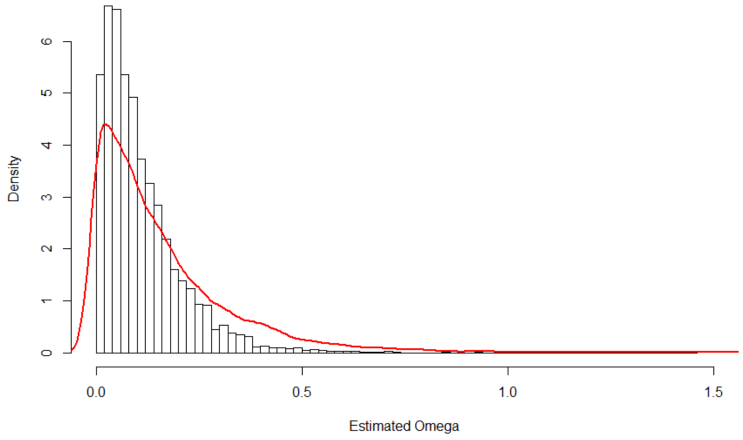

Supplementary Figure S5. Distribution of omega (d_N/d_S) values for 5,585 genes in the present study. Bars denote background (terrestrial mammals), and red lines denote the foreground branch (marine mammals).

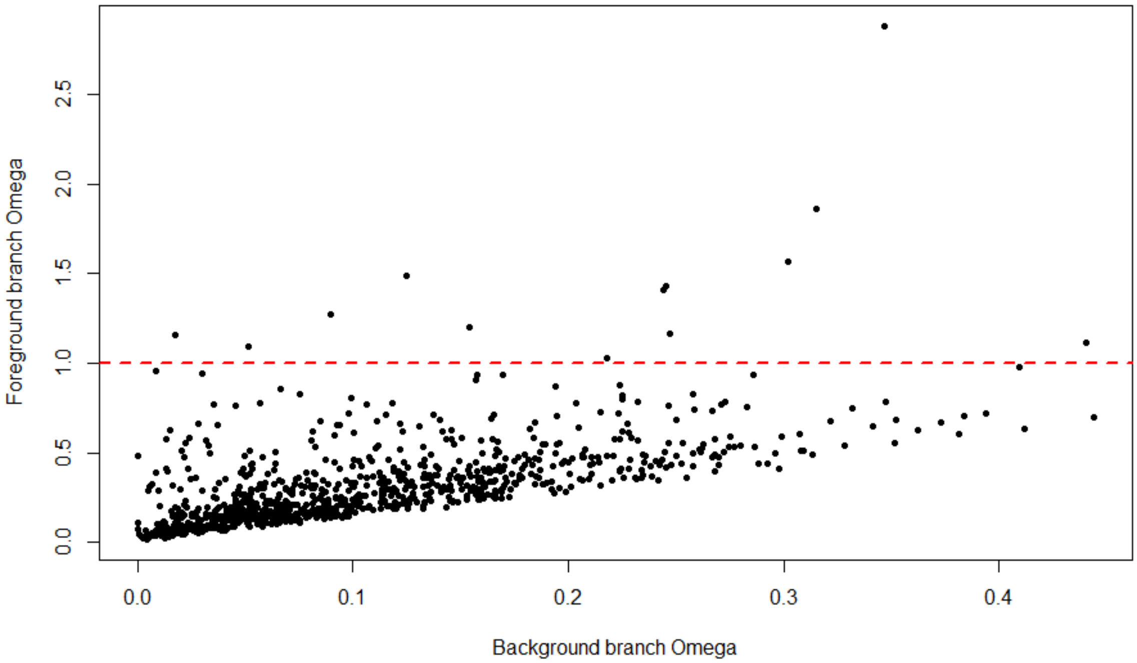

Supplementary Figure S6. Plot of omega (d_N/d_S) values for the 907 rapidly evolved genes in foreground branch (marine mammals) versus background branch (terrestrial mammals). Read line denotes the d_N/d_S values equal to one.

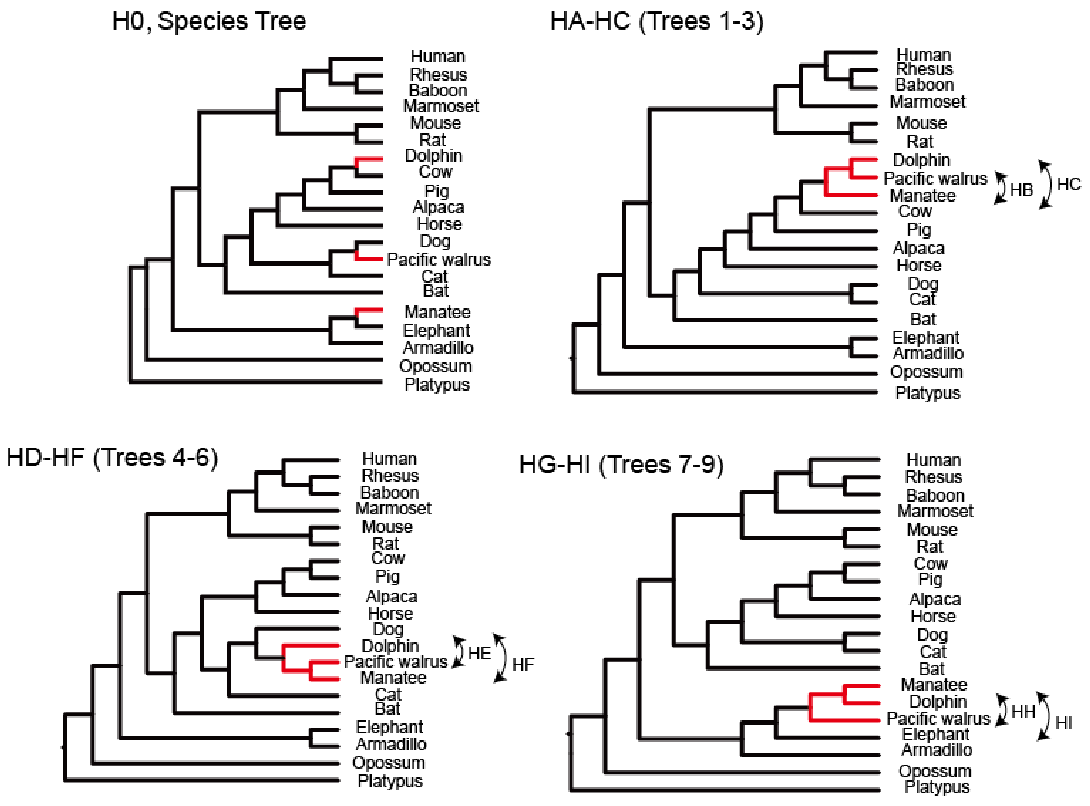

Supplementary Figure S7. Null hypothesis (species tree) and other 9 alternative hypotheses (Trees 1-9) examined in the likelihood convergence analyses.

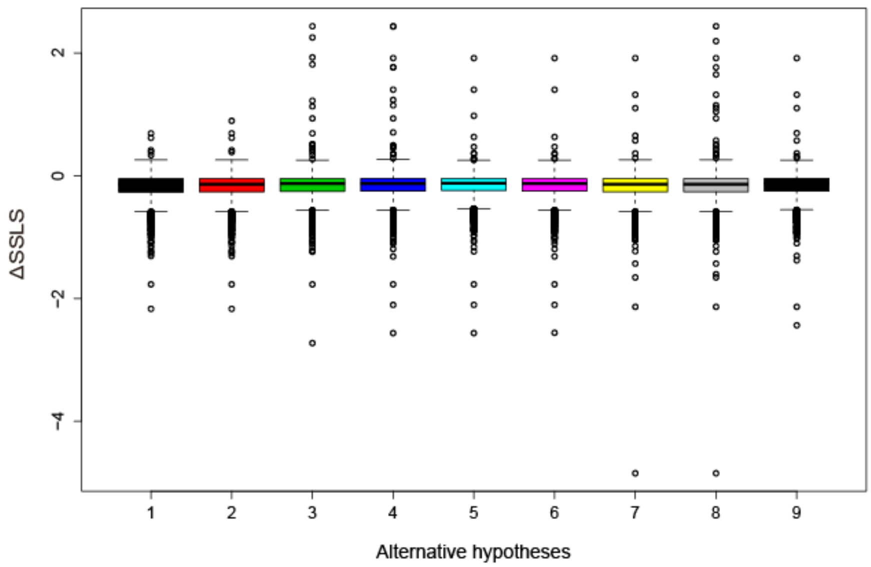

Supplementary Figure S8. Statistic of ΔSSLS values for 9 alternative topologies (Trees 1-9) which constraint the monophyly of marine mammals examined in the present study.

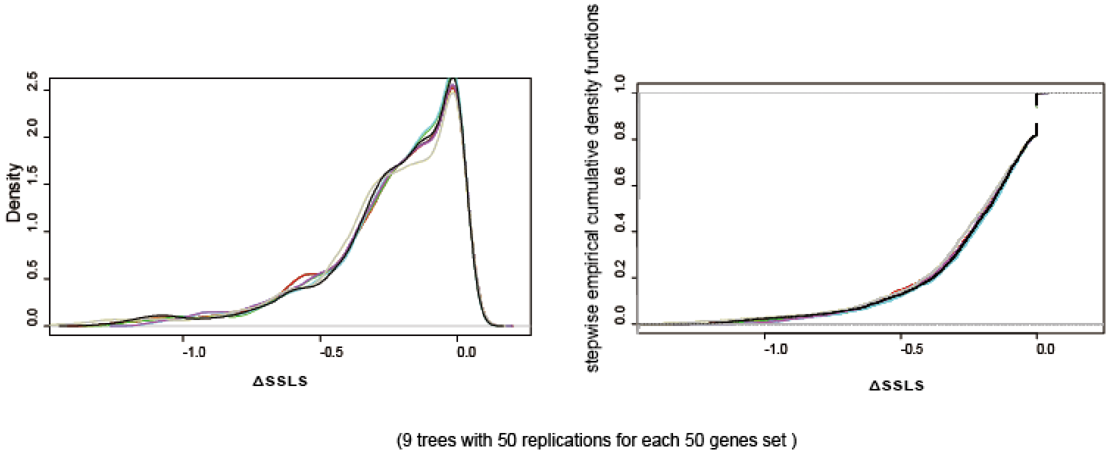

Supplementary Figure S9. Plots of ΔSSLS (left) and their stepwise empirical cumulative density functions (right) estimated from simulated data.

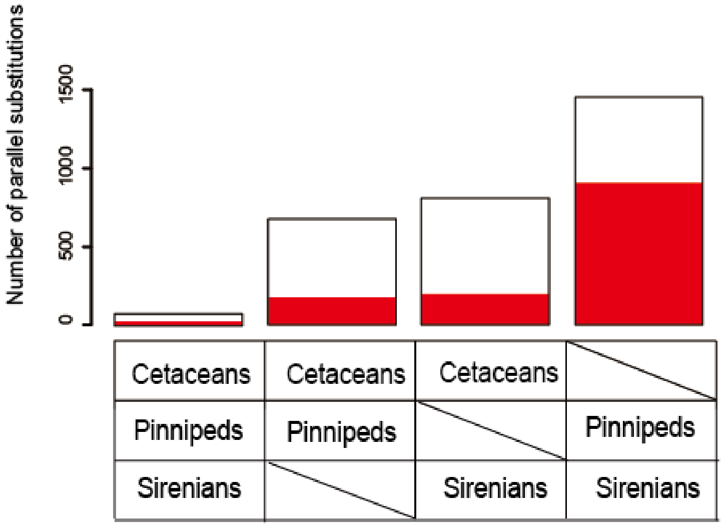

Supplementary Figure S10. Number of parallel substitutions in marine mammals identified in the alignment of orthologs from 20 mammals. Unique parallel substitutions are shaded in red.

Supplementary Tables

Supplementary Table S1. List of 133 parallel substitutions in the three marine lineages identified in the UCSC 100-way alignment.

RefGenes	Symbol	Position	Parallel substitution in marine mammals[a]
NM_001271208	NEB	3855	V-->I
NM_001080491	USP6NL	510	T-->I
NM_001145728	LMNTD1	377	A-->T
NM_001198806	MSANTD3	113	S-->N
NM_018012	KIF26B	261	V-->I
NM_003632	CNTNAP1	1040	I-->V
NM_000371	TTR	41	R-->Q
NM_194248	OTOF	1984	Y-->C
NM_001278370	MPP2	299	S-->N
NM_176895	PPAP2A	241	I-->V
NM_182641	BPTF	2125	T-->A
NM_203394	E2F7	675	V-->I
NM_020877	DNAH2	1650	M-->V
NM_001229	CASP9	88	L-->F
NM_012401	PLXNB2	878	N-->S
NM_144973	DENND5B	668	T-->A
NM_013365	GGA1	471	S-->G
NM_025251	ARHGAP39	259	P-->S
NM_001112734	ZSCAN30	112	R-->Q
NM_001146336	TMEM114	1	W-->X
NM_001198834	PDE4DIP	2105	P-->L
NM_021267	CERS1	181	V-->I
NM_178543	ENPP7	385	V-->I
NM_017617	NOTCH1	697	I-->V
NM_177531	PKHD1L1	2339	R-->Q
NM_001002294	FMO3	391	Q-->R
NM_001031720	GSTCD	171	T-->A
NM_001037163	FAM220A	142	R-->G
NM_001102416	KNG1	417	Q-->R
NM_021248	CDH22	620	A-->T
NM_002606	PDE9A	231	N-->S
NM_001085423	MILR1	1	W-->X
NM_001198956	DCAF6	492	A-->V
NM_033504	TMEM54	181	A-->T
NM_002114	HIVEP1	263	T-->A
NM_000393	COL5A2	253	A-->T
NM_001282281	PYCR1	301	Q-->R
NM_006424	SLC34A2	479	N-->S
NM_001113491	SEPT9	146	A-->T
NM_020884	MYH7B	1881	K-->Q
NM_032374	APOPT1	24	L-->F
NM_020869	DCDC5	148	H-->R
NM_020825	CRAMP1L	800	A-->T
NM_198098	AQP1	190	G-->S
NM_001278563	COL26A1	442	W-->X
NM_207468	FAM177B	15	G-->R
NM_024644	C14orf169	642	W-->X
NM_133475	ANKRD24	655	N-->S
NM_001098525	CKAP2	116	I-->V
NM_000694	ALDH3B1	469	W-->X

NM_001145512	NFIA	508	T-->A
NM_183008	UBXN11	132	M-->T
NM_001287390	CCBL1	26	T-->A
NM_015120	ALMS1	310	H-->R
NM_001077182	FSCN2	207	R-->H
NM_014002	IKBKE	498	R-->K
NM_006478	GAS2L1	682	W-->X
NM_172195	EIF2B4	372	E-->K
NM_001172818	PGM1	49	E-->A
NM_001278525	ZNF518A	1484	W-->X
NM_024032	C17orf53	295	R-->H
NM_001166663	CD244	86	T-->I
NM_002457	MUC2	283	G-->R
NM_002355	M6PR	130	N-->S
NM_032656	DHX37	1024	T-->A
NM_080744	SSC4D	512	R-->H
NM_001242409	GAREM	756	K-->E
NM_001385	DPYS	498	V-->I
NM_198534	C19orf45	481	H-->R
NM_001256041	MYO1A	492	R-->H
NM_001099626	MTFR1L	7	N-->S
NM_007300	BRCA1	1628	V-->A
NM_004727	SLC24A1	763	A-->T
NM_001199824	SBK3	360	W-->X
NM_024100	WDR18	381	A-->G
NM_001278211	LRRC20	80	R-->H
NM_001198934	ABCC10	57	D-->G
NM_031951	WDR87	372	Q-->R
NM_001802	CDR2	365	K-->R
NM_003613	CILP	868	R-->Q
NM_178173	CCDC36	564	N-->S
NM_175875	SIX5	630	T-->A
NM_138420	AHNAK2	5721	P-->L
NM_001110199	SRRM3	654	W-->X
NM_001198759	LY75-CD302	1137	H-->Q
NM_024552	CERS4	191	I-->V
NM_001200049	CFAP46	2572	R-->G
NM_000120	EPHX1	390	M-->I
NM_152354	ZNF285	311	S-->L
NM_018414	ST6GALNAC1	133	M-->V
NM_001001419	SMAD5	466	W-->X
NM_007289	MME	117	V-->I
NM_021185	CATSPERG	560	I-->V
NM_001172437	PEG10	1	W-->X
NM_015021	ZNF292	869	R-->G
NM_015909	NBAS	1390	A-->V
NM_138402	SP140L	368	M-->I
NM_001010879	ZIK1	107	P-->S
NM_002349	LY75	1137	H-->Q
NM_201266	NRP2	671	S-->G
NM_001286441	EXD1	498	S-->N
NM_002115	HK3	816	P-->S
NM_203437	AFTPH	54	R-->H
NM_005449	FAIM3	33	I-->V
NM_001198812	MSANTD3-TMEFF1	113	S-->N
NM_152513	MEI1	191	N-->S
NM_032534	KRBA1	246	R-->G
NM_001163629	MROH9	714	N-->S
NM_006184	NUCB1	14	P-->S
NM_001105520	C17orf100	119	W-->X
NM_018441	PECR	194	V-->I
NM_000488	SERPINC1	430	N-->S
NM_001080426	DUSP27	830	N-->S
NM_002473	MYH9	1650	Y-->C

NM_001167740	SMYD3	311	A-->T
NM_000616	CD4	448	P-->S
NM_001145051	TMEM247	220	W-->X
NM_001009909	LUZP2	102	S-->T
NM_003638	ITGA8	803	H-->R
NM_001134363	RBM20	745	P-->L
NM_006005	WFS1	109	H-->R
NM_006005	WFS1	606	V-->I
NM_170601	SIAE	468	I-->V
NM_000183	HADHB	128	K-->R
NM_181352	ATF7IP	936	T-->A
NM_052859	RFT1	502	I-->V
NM_001100112	MYH2	1792	M-->L
NM_001281740	FHOD3	695	A-->V
NM_001195833	RINL	328	G-->E
NM_001270424	DHRS12	171	I-->V
NM_016224	SNX9	132	A-->T
NM_213596	FOXN4	487	A-->T
NM_021925	C2orf43	36	N-->S

a, Parallel amino acid change in marine mammals (shown are Node of marine mammals and each terrestrial taxa --> marine mammals).

Supplementary Table S2. List of 26 genes with unique amino acid changes identified from the analysis of 65 mammalian genomes.

Symbol	RefGene	Terrestrial mammals[a]	Marine mammals[b]	Position	Unique amino acid change[c]	Parallel amino acid change[d]
CPT2	NM_000098	50/57	8/8	37	R --> LLLLLLLS	
TGM1	NM_000359	53/57	8/8	34	R --> HHHHHGGH	
GPAA1	NM_003801	54/57	8/8	421	G --> RRRRRTTD	
BST2	NM_004335	36/57	5/8	176	S --> ---EEIIM	
INSL5	NM_005478	49/57	7/8	119	T --> K-KKKRRV	
TBL1X	NM_005647	25/57	5/8	28	F --> ---PPPPW	
ZC3H7B	NM_017590	52/57	8/8	224	T --> PPPPPPPS	
ZBTB21	NM_020727	51/57	5/8	376	V --> ---SSGGG	
LMAN1L	NM_021819	56/57	8/8	257	P --> AAAAALLL	
WDR18	NM_024100	51/57	8/8	381	A --> RGRGGGGG	AAA -->GGGGG
ANKRD23	NM_144994	53/57	5/8	265	Y--> ---SSCWC	
SPATA2L	NM_152339	48/57	7/8	401	A --> -HHHHGGQ	
TMC8	NM_152468	44/57	8/8	68	R --> HHHHHSSL	
KNDC1	NM_152643	48/57	8/8	872	R --> HHHHHHHS	
KCNG4	NM_172347	56/57	8/8	163	R --> QQQQQGGQ	
TAS2R38	NM_176817	55/57	5/8	39	V --> ---MMMML	
SPNS3	NM_182538	48/57	7/8	27	Q --> PP-PPPPH	
ANXA2R	NM_001014279	23/57	5/8	159	L --> ---EEVVC	
SBK2	NM_001101401	47/57	8/8	198	R --> WWWWWWWG	

NLRP7	NM_001127255	34/57	8/8	811	K --> SSSSSMMM	
CCDC168	NM_001146197	46/57	5/8	3821	R --> ---TTEET	
DCAF6	NM_001198956	47/57	8/8	492	A --> VVVVVVVV	AAA -->VVVVV
MYBPC1	NM_001254718	57/57	7/8	404	G --> -EEEEEED	
NEIL1	NM_001256552	50/57	8/8	439	T --> RRRCCRRR	
C9orf173	NM_001256699	34/57	5/8	317	A --> ---SSSLQ	
OBSCN	NM_001271223	54/57	8/8	6674	S --> NCNNNDDA	

a, The number of terrestrial mammals which have the amino acid residue in the analyzed position/ the total number of terrestrial mammals. The remaining terrestrial mammals have gap at that position.

b, The number of marine mammals which have the amino acid residue in the analyzed position/the total number of marine mammals. The remaining marine mammals have gap at that position.

c, Unique amino acid change in marine mammals (shown in this order: Human --> Minke whale, Yangtze River dolphin, Sperm whale, Dolphin, Killer whale, Walrus, Weddell seal, Manatee). Dash denotes gap in the position of corresponding species.

d, Parallel amino acid change in marine mammals (shown are Node of cetaceans and Ruminantia, node of pinnipeds and panda, node of sirenians, elephant and Cape elephant shrew --> Dolphin, Killer whale, Walrus, Weddell seal, Manatee).

Supplementary Table S3. List of 70 aquatic adaptation genes identified in the three marine mammalian lineages. ΔSSLS with significant statistical support are shaded in gray.

Symbol	dN/dS (Background)	dN/dS (Marine mammals)	P (LRT)	ΔSSLS & P value (From convergence hypotheses HA to HI)																	
				HA	P(cdf)	HB	P(cdf)	HC	P(cdf)	HD	P(cdf)	HE	P(cdf)	HF	P(cdf)	HG	P(cdf)	HH	P(cdf)	HI	P(cdf)
M6PR	0.072	0.283	0.019	0.0027	0.007	0.0027	0.007	0.0027	0.007	-0.0449	0.239	-0.0027	0.184	-0.0449	0.238	0.0027	0.004	0.0027	0.005	0.0027	0.005
ERLEC1	0.044	0.175	0.018	0.0266	0.002	0.0266	0.002	0.0266	0.002	0.0266	0.002	0.0266	0.002	0.0266	0.002	0.0070	0.002	0.0198	0.001	0.0070	0.003
RHOV	0.018	0.137	0.028	0.0000	0.155	0.0000	0.155	0.0000	0.159	0.0000	0.136	0.0000	0.148	0.0000	0.141	0.0000	0.010	0.0000	0.012	0.0000	0.044
CKM	0.033	0.094	0.007	-0.0198	0.196	-0.0180	0.196	-0.0233	0.202	0.0167	0.003	0.0222	0.003	0.0195	0.003	0.0496	0.000	0.0550	0.000	0.0465	0.000
CTSC	0.189	0.547	0.001	-0.3632	0.762	-0.3632	0.762	0.5162	0.000	0.4870	0.000	-0.0906	0.337	-0.0906	0.326	-0.3242	0.703	0.4395	0.000	-0.3242	0.736
RPL22	0.010	0.077	0.017	0.0000	0.160	0.0000	0.160	0.0000	0.160	0.0000	0.048	0.0000	0.043	0.0000	0.044	0.0000	0.149	0.0000	0.151	0.0000	0.152
ATG14	0.062	0.234	0.015	0.0134	0.005	0.0134	0.005	0.0214	0.003	-0.0195	0.198	-0.0275	0.214	-0.0275	0.213	0.0381	0.001	0.0381	0.001	0.0381	0.001
CKMT2	0.023	0.112	0.000	0.0000	0.010	-0.0123	0.010	-0.0043	0.186	-0.0043	0.185	-0.0123	0.191	0.0000	0.009	-0.0178	0.194	-0.0098	0.188	-0.0055	0.185
PHC2	0.060	0.174	0.001	-0.0027	0.184	0.0016	0.184	-0.0114	0.190	-0.0079	0.188	0.0050	0.005	0.0008	0.008	0.0160	0.001	0.0031	0.005	0.0117	0.002
DDX25	0.043	0.123	0.020	0.0001	0.010	0.0001	0.010	0.0001	0.011	-0.0027	0.183	-0.0027	0.183	-0.0027	0.183	0.0184	0.001	0.0184	0.001	0.0184	0.002
AGPAT9	0.078	0.314	0.000	-0.0008	0.181	-0.0008	0.181	-0.0008	0.181	-0.0127	0.191	-0.0127	0.191	-0.0127	0.190	0.0062	0.002	0.0062	0.003	0.0062	0.003
GRIA2	0.054	0.184	0.000	-0.1744	0.485	-0.1744	0.485	0.0150	0.004	0.0150	0.004	0.0097	0.005	0.0097	0.005	-0.1610	0.436	0.0150	0.002	-0.1610	0.471
MAP3K13	0.045	0.177	0.000	0.0000	0.144	0.0000	0.144	0.0000	0.144	0.0000	0.022	0.0000	0.028	0.0000	0.026	0.0000	0.023	0.0000	0.019	0.0000	0.028
SAE1	0.089	0.328	0.000	-0.1189	0.380	-0.1189	0.380	0.1705	0.000	0.1423	0.000	-0.1370	0.439	-0.1370	0.415	-0.1493	0.417	0.1205	0.000	-0.1449	0.438
KLHL8	0.032	0.137	0.001	0.0029	0.007	0.0029	0.007	0.0029	0.007	-0.0131	0.191	-0.0131	0.191	-0.0131	0.190	0.0029	0.004	0.0029	0.005	0.0029	0.005
LIN7C	0.002	0.031	0.014	0.0000	0.042	0.0000	0.042	0.0000	0.044	0.0000	0.041	0.0000	0.035	0.0000	0.044	0.0000	0.168	0.0000	0.169	0.0000	0.031

THRB	0.235	0.488	0.000	0.4158	0.000	0.4151	0.000	0.4175	0.000	0.4685	0.000	0.4685	0.000	0.4691	0.000	-0.1240	0.373	0.3671	0.000	-0.1240	0.394
PDLIM3	0.138	0.373	0.000	0.0245	0.002	0.0205	0.002	0.0314	0.002	-0.0521	0.254	-0.4949	0.877	-0.3951	0.800	-0.0685	0.277	-0.1093	0.346	-0.0187	0.196
AIFM1	0.066	0.345	0.000	-0.0263	0.206	-0.0263	0.206	0.4507	0.000	0.4507	0.000	-0.0263	0.212	-0.0377	0.226	-0.0338	0.217	0.4269	0.000	-0.0267	0.208
CCNYL1	0.072	0.265	0.014	-0.0016	0.182	-0.0016	0.182	0.2545	0.000	0.2545	0.000	-0.0016	0.181	-0.0190	0.198	-0.0016	0.181	0.2545	0.000	-0.0060	0.185
CCNL1	0.014	0.394	0.000	0.0000	0.043	0.0000	0.043	0.0000	0.043	0.0000	0.040	0.0000	0.038	0.0000	0.044	0.0000	0.032	0.0000	0.034	0.0000	0.037
LRFN5	0.014	0.081	0.006	0.0000	0.044	0.0000	0.044	0.0000	0.033	0.0000	0.037	0.0000	0.026	0.0000	0.033	0.0000	0.024	0.0000	0.032	0.0000	0.022
SEC23A	0.013	0.090	0.000	0.0000	0.034	0.0000	0.034	0.0000	0.024	0.0000	0.019	0.0000	0.019	0.0000	0.039	0.0000	0.010	0.0000	0.009	0.0000	0.020
HNRNPD	0.000	0.109	0.001	-0.0013	0.182	-0.0013	0.182	0.0174	0.003	0.0174	0.003	-0.0013	0.181	-0.0013	0.181	-0.0013	0.181	0.0095	0.002	-0.0013	0.182
FGF13	0.052	0.381	0.000	0.2253	0.000	0.2253	0.000	0.2281	0.000	0.2281	0.000	0.2253	0.000	0.2253	0.000	0.2253	0.000	0.2281	0.000	0.2253	0.000
PABPC1	0.018	0.097	0.000	0.0000	0.053	0.0000	0.053	0.0000	0.052	0.0000	0.051	0.0000	0.050	0.0000	0.052	0.0000	0.037	0.0000	0.042	0.0000	0.038
GLS	0.063	0.401	0.000	0.3326	0.000	0.0000	0.000	0.0000	0.146	0.3637	0.000	0.3637	0.000	0.3637	0.000	0.3637	0.000	0.3637	0.000	0.3637	0.000
DSCAML1	0.030	0.943	0.013	0.0003	0.010	0.0003	0.010	0.0003	0.011	0.0003	0.008	0.0003	0.008	0.0003	0.008	0.0000	0.138	0.0000	0.140	0.0000	0.136
ANXA2	0.035	0.110	0.002	0.0115	0.005	0.0202	0.005	0.0115	0.004	-0.0123	0.191	-0.0037	0.185	-0.0123	0.189	0.0199	0.001	0.0128	0.002	0.0128	0.002
ASB7	0.011	0.041	0.047	0.0000	0.040	0.0000	0.040	0.0000	0.038	0.0000	0.033	0.0000	0.033	0.0000	0.036	0.0000	0.027	0.0000	0.028	0.0000	0.028
SF3B1	0.004	0.065	0.027	0.0650	0.000	0.0650	0.000	2.4342	0.000	2.4342	0.000	0.0650	0.000	0.0650	0.000	0.0650	0.000	2.4342	0.000	0.0650	0.000
BCL11A	0.166	0.405	0.002	0.3843	0.000	0.3843	0.000	0.3843	0.000	0.3484	0.000	0.0375	0.002	0.0375	0.002	0.0375	0.001	0.3484	0.000	0.0375	0.001
SPTY2D1	0.315	1.862	0.020	0.2124	0.000	0.2124	0.000	0.2124	0.000	0.2124	0.000	0.2124	0.000	0.2124	0.000	0.2124	0.000	0.2124	0.000	0.2124	0.000
SH2D4B	0.114	0.295	0.000	-0.2007	0.525	-0.2007	0.525	0.0696	0.000	0.0767	0.000	-0.1532	0.472	-0.1532	0.449	-0.1547	0.425	0.0681	0.000	-0.1547	0.457
RNF13	0.053	0.132	0.018	-0.0261	0.206	-0.0257	0.206	-0.0307	0.213	-0.0283	0.213	-0.0098	0.189	-0.0226	0.205	0.0053	0.002	0.0002	0.006	0.0002	0.006
MYL1	0.035	0.127	0.044	-0.0993	0.336	-0.0480	0.336	-0.0993	0.349	-0.0529	0.254	0.0183	0.004	-0.0529	0.254	-0.0061	0.185	-0.0373	0.224	-0.0373	0.226
ST5	0.012	0.081	0.002	-0.0039	0.185	-0.0039	0.185	-0.0039	0.186	-0.0018	0.182	0.0084	0.005	-0.0018	0.182	-0.0077	0.187	-0.0121	0.189	-0.0121	0.188
MAPK10	0.015	0.076	0.000	0.0000	0.053	0.0000	0.053	0.0000	0.024	0.0000	0.051	0.0000	0.028	0.0000	0.139	0.0000	0.139	0.0000	0.034	0.0000	0.136
ESRRG	0.007	0.054	0.000	0.0000	0.028	0.0000	0.028	0.0000	0.027	0.0000	0.033	0.0000	0.035	0.0000	0.033	0.0000	0.044	0.0000	0.044	0.0000	0.034

EPHA7	0.037	0.071	0.016	-0.0222	0.199	-0.0222	0.199	0.0118	0.004	0.0127	0.004	-0.0164	0.195	-0.0164	0.194	-0.0160	0.191	0.0127	0.002	-0.0164	0.192
EPS15	0.098	0.721	0.000	0.0311	0.002	0.0311	0.002	0.9332	0.000	0.9332	0.000	0.0311	0.002	0.0311	0.002	0.0311	0.001	0.9332	0.000	0.0311	0.001
GLRA4	0.055	0.134	0.014	-0.0105	0.190	-0.0105	0.190	-0.0105	0.190	0.0040	0.005	0.0040	0.005	0.0040	0.005	0.0040	0.003	0.0040	0.004	0.0040	0.004
RBBP4	0.024	0.093	0.005	0.0000	0.016	0.0000	0.016	0.0000	0.060	0.0000	0.054	0.0000	0.054	0.0000	0.013	0.0000	0.054	0.0000	0.054	0.0000	0.009
PARK7	0.122	0.344	0.007	0.0103	0.005	0.0178	0.005	0.0103	0.004	0.0103	0.004	0.0178	0.004	0.0103	0.004	0.0159	0.002	0.0159	0.002	0.0159	0.002
PLCB4	0.032	0.092	0.000	-0.1495	0.444	-0.1495	0.444	-0.0704	0.290	-0.0728	0.288	-0.1262	0.413	-0.1263	0.392	0.0100	0.002	0.0100	0.002	0.0170	0.002
GALT	0.073	0.326	0.000	-0.0352	0.220	-0.0352	0.220	0.2048	0.000	0.2048	0.000	-0.0352	0.224	-0.0352	0.222	-0.0352	0.220	0.2048	0.000	-0.0352	0.221
NPSR1	0.057	0.778	0.000	0.0047	0.006	0.0047	0.006	0.0047	0.006	-0.0352	0.222	-0.0352	0.224	-0.0352	0.222	0.0047	0.002	0.0047	0.003	0.0047	0.003
MB21D2	0.004	0.019	0.013	0.0000	0.042	0.0000	0.042	0.0000	0.040	0.0000	0.036	0.0000	0.035	0.0000	0.043	0.0000	0.031	0.0000	0.032	0.0000	0.029
MEIS1	0.125	1.485	0.000	0.0000	0.060	0.0000	0.060	0.0000	0.012	0.0000	0.010	0.0000	0.054	0.0000	0.054	0.0000	0.054	0.0000	0.008	0.0000	0.052
ZSWIM8	0.112	0.539	0.000	-0.0357	0.221	-0.0357	0.221	0.4954	0.000	0.4954	0.000	-0.0357	0.225	-0.0357	0.223	-0.0357	0.222	0.4954	0.000	-0.0357	0.223
ROCK1	0.017	0.193	0.006	0.0000	0.144	0.0000	0.144	0.0000	0.060	0.0000	0.034	0.0000	0.033	0.0000	0.139	0.0000	0.037	0.0000	0.037	0.0000	0.029
SZRD1	0.224	0.881	0.027	0.0000	0.148	0.0000	0.148	0.0000	0.149	0.0000	0.044	0.0000	0.035	0.0000	0.044	0.0000	0.018	0.0000	0.141	0.0000	0.012
AGR2	0.118	0.293	0.005	-0.0716	0.286	-0.0716	0.286	-0.0716	0.292	0.0258	0.002	0.0258	0.002	0.0258	0.002	-0.0119	0.189	-0.0119	0.189	-0.0119	0.188
FAM49B	0.012	0.115	0.000	-0.0123	0.190	-0.0123	0.190	0.0791	0.000	0.0791	0.000	-0.0123	0.191	-0.0123	0.189	-0.0123	0.189	0.0791	0.000	-0.0123	0.188
PRKCA	0.017	0.059	0.006	0.0064	0.005	0.0064	0.005	0.0064	0.005	0.0064	0.005	0.0064	0.005	0.0064	0.005	-0.0316	0.212	-0.0316	0.212	-0.0263	0.207
LDB3	0.193	0.271	0.000	0.6157	0.000	0.6157	0.000	1.1291	0.000	1.2349	0.000	0.6312	0.000	0.6312	0.000	0.6526	0.000	1.0426	0.000	0.6933	0.000
YBX1	0.022	0.556	0.000	0.0000	0.155	0.0000	0.155	0.0000	0.156	0.0000	0.050	0.0000	0.034	0.0000	0.050	0.0000	0.034	0.0000	0.035	0.0000	0.034
HERPUD1	0.093	0.653	0.000	-0.0863	0.313	-0.0863	0.313	0.3326	0.000	0.2624	0.000	-0.1378	0.441	-0.1562	0.454	-0.0863	0.306	0.3326	0.000	-0.0863	0.314
STK36	0.019	0.148	0.003	0.0000	0.055	0.0000	0.055	0.0000	0.057	0.0000	0.054	0.0000	0.050	0.0000	0.054	0.0000	0.041	0.0000	0.144	0.0000	0.050
TSC22D1	0.154	1.202	0.007	0.2561	0.000	0.2561	0.000	2.2508	0.000	2.4279	0.000	0.2550	0.000	0.2550	0.000	0.2549	0.000	2.1917	0.000	0.2549	0.000
PIGP	0.094	0.655	0.001	0.0030	0.007	0.0433	0.007	0.0030	0.007	0.0030	0.006	0.0433	0.002	0.0030	0.006	-0.0082	0.187	-0.0485	0.244	-0.0485	0.248
ADPRH	0.080	0.572	0.000	-0.0165	0.193	-0.0169	0.193	0.0003	0.011	-0.0554	0.259	-0.0411	0.236	-0.0708	0.286	-0.0563	0.254	-0.0750	0.288	-0.0880	0.317

GRIA1	0.020	0.092	0.030	0.0000	0.042	0.0000	0.042	0.0000	0.054	0.0000	0.048	0.0000	0.048	0.0000	0.036	0.0000	0.051	0.0000	0.050	0.0000	0.026
NRG1	0.149	0.369	0.000	0.1174	0.000	0.1174	0.000	0.5072	0.000	0.7041	0.000	0.2349	0.000	0.2349	0.000	0.1274	0.000	0.5064	0.000	0.1335	0.000
KCNS1	0.022	0.229	0.000	-0.0673	0.276	-0.0452	0.276	-0.0597	0.269	-0.0059	0.186	0.0087	0.005	-0.0059	0.185	-0.0319	0.212	-0.0465	0.239	-0.0446	0.239
NOVA1	0.010	0.202	0.000	0.0000	0.056	0.0000	0.056	0.0000	0.057	0.0000	0.053	0.0000	0.052	0.0000	0.053	0.0000	0.041	0.0000	0.042	0.0000	0.042
CACNB4	0.032	0.079	0.026	0.0000	0.041	0.0000	0.041	0.0000	0.033	0.0000	0.024	0.0000	0.028	0.0000	0.033	0.0000	0.034	0.0000	0.037	0.0000	0.034
PPFIA2	0.053	0.435	0.002	0.0000	0.155	0.0000	0.155	0.0000	0.148	0.0000	0.156	0.0000	0.048	0.0000	0.151	0.0000	0.041	0.0000	0.019	0.0000	0.137
STXBP6	0.060	0.183	0.007	0.0000	0.057	0.0000	0.057	0.0000	0.054	0.0000	0.034	0.0000	0.029	0.0000	0.051	0.0000	0.023	0.0000	0.052	0.0000	0.166
NOP16	0.052	0.231	0.014	-0.0182	0.194	0.0171	0.194	-0.0182	0.194	-0.0182	0.197	0.0171	0.004	-0.0182	0.197	0.0171	0.001	-0.0182	0.195	-0.0182	0.195

Notes:

dN/dS denotes the rate of protein evolution of marine and terrestrial mammals.

P (LRT), P value is based on LRT test.

ΔSSLS, Mean sitewise ΔSSLS for each gene; positive values imply support for an alternative hypothesis.

P (cdf), P is the significance of an observed site ΔSSLS comparison of the species topology and alternative hypothesis.

Supplementary Table S4. List of 70 aquatic adaptation genes with the number of unique amino acid changes in all three lineages of marine mammals (III), in any two of the three marine lineages (II) and in single lineages of marine mammals (I).

Symbol	I	II	III
M6PR	3		1
ERLEC1	3		
RHOV	3		
CKM	7		
CTSC	12	13	
RPL22	2		
ATG14	7		
CKMT2	10	2	
PHC2	13	1	
DDX25	7		
AGPAT9	58	1	
GRIA2	63	1	
MAP3K13	52		
SAE1	13	17	
KLHL8	16		
LIN7C	1		
THRB	6		

PDLIM3	12	2	
AIFM1	53	23	1
CCNYL1	56	9	
CCNL1	5		
LRFN5	4		
SEC23A	37		
HNRNPD		1	
FGF13	3		
PABPC1	10		
GLS	1		
DSCAML1	3		
ANXA2	9		
ASB7	4		
SF3B1	6	102	4
BCL11A	6		
SPTY2D1	3		1
SH2D4B	14	3	
RNF13	20		
MYL1		1	
ST5	56		

MAPK10	1	
ESRRG	4	
EPHA7	8	2
EPS15	1	15
GLRA4	21	1
RBBP4	77	2
PARK7	5	
PLCB4	43	1
GALT	4	5
NPSR1	51	5
MB21D2	4	
MEIS1	15	1
ZSWIM8	5	3
ROCK1	2	
SZRD1	7	
AGR2	6	
FAM49B	1	8
PRKCA	10	
LDB3	14	
YBX1	52	

HERPUD1	14	16	
STK36	1		
TSC22D1		1	
PIGP	29	3	
ADPRH	17	1	
GRIA1	18		
NRG1	9	32	1
KCNS1	85	2	
NOVA1	38		
CACNB4	43		
PPFIA2	4		
STXBP6	7		
NOP16	3	1	

Supplementary Table S5. Biological Process (BP) GO term enrichment result of 70 aquatic adaptation genes. Over-represented GO terms were defined as having Fisher exact test $P \leq 0.05$.

GO ID	Description	Count	%	P Value	Fold Enrichment	Fisher Exact Test P
GO:0007268	synaptic transmission	7	10.6	0.001	5.8	1.90E-04
GO:0048255	mRNA stabilization	3	4.5	0.002	49.2	2.80E-05
GO:0043489	RNA stabilization	3	4.5	0.002	49.2	2.80E-05
GO:0019226	transmission of nerve impulse	7	10.6	0.003	4.9	5.10E-04
GO:0043488	regulation of mRNA stability	3	4.5	0.003	33.5	9.30E-05
GO:0043487	regulation of RNA stability	3	4.5	0.004	30.7	1.20E-04
GO:0007267	cell-cell signaling	8	12.1	0.009	3.3	2.80E-03
GO:0010608	posttranscriptional regulation of gene expression	5	7.6	0.010	5.8	1.60E-03
GO:0004111	creatine kinase activity	2	3.0	0.016	124.8	9.40E-05
GO:0006898	receptor-mediated endocytosis	3	4.5	0.019	13.9	1.30E-03
GO:0000377	RNA splicing, via transesterification reactions with bulged adenosine as nucleophile	4	6.1	0.023	6.4	3.40E-03
GO:0000375	RNA splicing, via transesterification reactions	4	6.1	0.023	6.4	3.40E-03
GO:0000398	nuclear mRNA splicing, via spliceosome	4	6.1	0.023	6.4	3.40E-03
GO:0008380	RNA splicing	5	7.6	0.026	4.3	5.80E-03
GO:0003729	mRNA binding	3	4.5	0.028	11.3	2.30E-03
GO:0005230	extracellular ligand-gated ion channel activity	3	4.5	0.032	10.4	3.00E-03

GO:0022836	gated channel activity	5	7.6	0.033	4.0	7.80E-03
GO:0006600	creatine metabolic process	2	3.0	0.035	54.7	5.70E-04
GO:0006599	phosphagen metabolic process	2	3.0	0.035	54.7	5.70E-04
GO:0000165	MAPKKK cascade	4	6.1	0.037	5.3	6.60E-03
GO:0006397	mRNA processing	5	7.6	0.039	3.8	9.60E-03
GO:0031623	receptor internalization	2	3.0	0.043	44.7	8.70E-04
GO:0007242	intracellular signaling cascade	10	15.2	0.059	2.0	2.80E-02
GO:0016071	mRNA metabolic process	5	7.6	0.060	3.3	1.70E-02
GO:0005216	ion channel activity	5	7.6	0.064	3.2	1.90E-02
GO:0016775	phosphotransferase activity, nitrogenous group as acceptor	2	3.0	0.065	29.4	2.10E-03
GO:0030295	protein kinase activator activity	2	3.0	0.068	27.7	2.30E-03
GO:0004970	ionotropic glutamate receptor activity	2	3.0	0.068	27.7	2.30E-03
GO:0022838	substrate specific channel activity	5	7.6	0.070	3.1	2.10E-02
GO:0005234	extracellular-glutamate-gated ion channel activity	2	3.0	0.072	26.3	2.60E-03
GO:0043167	ion binding	23	34.8	0.076	1.4	5.40E-02
GO:0015267	channel activity	5	7.6	0.078	3.0	2.40E-02
GO:0022803	passive transmembrane transporter activity	5	7.6	0.078	3.0	2.40E-02
GO:0016192	vesicle-mediated transport	6	9.1	0.079	2.6	2.90E-02
GO:0004674	protein serine/threonine kinase activity	5	7.6	0.088	2.9	2.80E-02
GO:0004672	protein kinase activity	6	9.1	0.088	2.5	3.30E-02

Supplementary Table S6. KEGG Pathway enrichment of 70 aquatic adaptation genes. Over-represented GO terms were defined as having Fisher exact test $P \leq 0.05$.

KEGG ID	Description	Count	%	P Value	Fold Enrichment	Fisher Exact Test P
hsa04720	Long-term potentiation	4	6.1	0.004	11.5	3.50E-04
hsa04730	Long-term depression	4	6.1	0.004	11.3	3.70E-04
hsa00330	Arginine and proline metabolism	3	4.5	0.027	11.1	2.30E-03
hsa04310	Wnt signaling pathway	4	6.1	0.037	5.2	6.70E-03
hsa04010	MAPK signaling pathway	5	7.6	0.039	3.7	1.00E-02
hsa04012	ErbB signaling pathway	3	4.5	0.067	6.7	9.50E-03
hsa04912	GnRH signaling pathway	3	4.5	0.083	6.0	1.30E-02
